# Diurnal Patterns of Physical Activity in Relation to Activity Induced Energy Expenditure in 52 to 83 Years-Old Adults

**DOI:** 10.1371/journal.pone.0167824

**Published:** 2016-12-09

**Authors:** Giulio Valenti, Alberto G. Bonomi, Klaas R. Westerterp

**Affiliations:** 1 Department of Human Biology, Maastricht University, Maastricht, The Netherlands; 2 Personal Health Solutions, Philips Research Laboratories, Eindhoven, The Netherlands; Vanderbilt University, UNITED STATES

## Abstract

**Background:**

Ageing is associated with a declining physical activity level (PAL) and changes in the diurnal activity pattern. Changes in the activity pattern might help explaining the age-associated reduction of physical activity.

**Objective:**

The aims were to investigate diurnal activity patterns within groups of older adults classified by PAL, to investigate diurnal activity patterns within age-groups and to investigate the association between the drop in activity and aerobic fitness.

**Methods:**

Thirty-one healthy subjects aged between 52 and 83y were recruited for the study. Subjects were divided in sedentary (PAL<1.75), moderately active (1.75<PAL<1.90), and active (1.90<PAL) older adults with energy expenditure measurements obtained with the doubly labelled water technique. Diurnal activity patterns were based on activity counts from an accelerometer during wake time and then divided in four quarters of equal time length. Additionally, aerobic fitness was measured as maximal oxygen uptake.

**Results:**

Subjects had a PAL between 1.43 and 2.34 and an aerobic fitness between 18 and 49 ml/kg/min. Overall, activity patterns showed a peak in the first quarter of wake time (around 10AM) followed by a gradual decline of, on average, 5% per hour. Active subjects reached their peak in the first quarter and remained active until after the third quarter (11% drop each quarter on average). Moderately active and sedentary subjects reached their peak during the second quarter with a decrease during the third quarter (respectively 29% and 17% drop each quarter on average). The drop in physical activity between the first and the second half of the wake time was negatively associated with aerobic fitness (r = -0.39, p<0.05).

**Conclusion:**

Active older adults maintained a larger amount of body movement for longer during their wake time. Diurnal physical activity declined more in adults ≥66 years old with lower aerobic fitness.

## Introduction

Increasing age is accompanied by a gradual decline of physical activity and an increase in sedentary behaviour after the age of 50 [1, 2]. A sedentary lifestyle in older adults is associated with several negative health outcomes such as disability [3], sarcopenia [4], diabetes, [5] cardiovascular diseases [6] and increased morbidity [7]. Therefore, the World Health Organization sets specific goals of time spent in moderate to vigorous physical activity physical activity, namely 150 min/week in bouts longer than 10 minutes for individuals after the 65th year of age [8].

Objective assessments of physical activity level (PAL) in daily-living can be carried out by measuring total energy expenditure with the doubly labelled water technique (DLW) or by means of accelerometers [9]. The DLW is a highly accurate method to measure total energy expenditure and derive PAL in daily life (PAL = total energy expenditure / resting energy expenditure) [10]. The technique based on DLW provides the average PAL over the period of measure with no information about physical activity patterns. Accelerometers provide less accurate assessments of PAL than the DLW technique, but they preserve the information on activity patterns [9]. Therefore, combining measurements of PAL from DLW with activity patterns as provided by accelerometers can reveal differences in activity patterns between subjects with different PAL.

Diurnal activity patterns have been objectively characterized before to describe how physical activity is distributed during the day. Physical activity reaches a peak between 10 and 11AM, after which it declines, allowing a gradual increase in sedentary behaviours [11, 12]. Previous studies also demonstrated that with increasing age diurnal activity peaks disappeared with larger decreases in late afternoon and evening [13]. It has been therefore suggested that prolonging active morning bouts of physical activity could contrast the decline in physical activity and it might be an effective strategy to maintain higher PAL [11]. However, studies about physical activity patterns have never classified subjects according to their energy-expenditure derived PAL and there is no evidence so far that subjects with higher PAL are less prone to the diurnal decline in physical activity.

Increasing age and reduced physical activity are associated with declining aerobic fitness [14, 15], measured as maximal aerobic capacity of an individual [16, 17]. Aerobic fitness has been described both as a determinant and as an outcome of physical activity [18]. Aerobic fitness can determine physical activity; subjects with a low aerobic capacity are more prone to fatigue [19, 20] and their endurance is compromised [21]. Increased fatigue reported in unfit individuals might discourage them from being physical active, especially later in the day. At the same time, physical activity interventions such as endurance training [22] or lifestyle interventions [23] have been shown to increase aerobic capacity, indicating that improved aerobic fitness is one of the outcomes of increased physical activity. Thus, aerobic fitness is a potential covariate in studies concerning physical activity. Previous studies where aerobic fitness and physical activity were measured have not discriminated physical activity by time of the day, thus the relationship between aerobic fitness and diurnal activity patterns has not been reported yet.

The design of intervention strategies for increasing physical activity to promote healthy ageing might benefit from more insights into how diurnal activity pattern characterizes older adults with a higher PAL. The aims were to investigate diurnal activity patterns within groups of older adults classified by PAL, to investigate diurnal activity patterns within age-groups and to investigate the association between the drop in activity and aerobic fitness.

## Methods

### Population

Thirty-one healthy subjects aged between 52 and 83y (BMI 25.7±2.1 kg/m2) were recruited by advertisements in local newspapers. Respondents signed a written informed consent and completed a questionnaire including metabolic or orthopaedic conditions, neurological disorders and cardiovascular problems. A doctor discussed the questionnaire with the respondents during a medical visit. All subjects were in good orthopaedic, neurological and cardiovascular conditions and were included. At the end of the study no subject reported onset signs of metabolic or orthopaedic conditions, neurological disorders and cardiovascular problems. The study was conducted according to the Declaration of Helsinki and the Ethics Committee of the Maastricht University Medical Center approved the study. This trial was registered at www.clinicaltrials.gov as NCT01609764.

### Study design

The study followed a cross-sectional design. Basal metabolic rate was measured on the first morning. Subsequently, free-living energy expenditure was measured in daily-life. Physical activity was monitored simultaneously with an accelerometer, and subjects reported wake-up and go-to-bed times in a diary. Aerobic fitness was measured at least two days before or after the physical activity measurement.

### Energy expenditure

Basal metabolic rate (BMR) was measured during 30 minutes under a ventilated hood in the supine position under standard conditions of rest, fasting, immobility, thermoneutrality and mental relaxation [24]. Oxygen consumption and carbon dioxide production sampled from the hood were measured with an indirect calorimeter (Omnical, Maastricht Instruments, Maastricht, The Netherlands). Basal metabolic rate was calculated from O2 consumption and CO2 production with Brouwer’s formula [25].

Total energy expenditure (TEE) was measured using doubly labelled water, as previously described [26]. Briefly, after the collection of a baseline urine sample on the evening of day 0, subjects drank a weighted amount of 2H218O. The dose resulted in a body water enrichment of about 120 p.p.m. for 2H and about 240 p.p.m. for 18O. Urine samples were collected from the second voiding in the mornings of days 1, 8 and 15, and in the evenings of days 1, 7 and 14. Urine samples were analysed with an isotope ratio mass spectrometer (Optima; VG Isogas, Middlewich, Cheshire, UK). Carbon dioxide production was calculated from the difference between the elimination rates of 2H and 18O. Total daily energy expenditure was calculated from carbon dioxide production, assuming a respiratory quotient of 0.85 [27].

### Physical activity

Subjects wore a tri-axial accelerometer (GT3X+, ActiGraph, Pensacola, FL) during 2 weeks on the lower back using a belt, as described before [28]. Subjects were instructed to wear the accelerometer on the lower back from wake-up until go-to-bed time and to report the time in a diary. Non-wearing events included water activities, such as bathing or showering, and were reported in the same diary to allow the calculation of wearing time. Accelerometer data were exported to Matlab (Mathworks, Natick, MA) and divided in epochs of 5s. Counts were calculated by integrating body accelerations of each epoch after detrending and rectification of the signal from each axis. Activity counts were then integrated in epochs of one minute. Data during non-wearing time was imputed zero counts. Days during which data were missing or subjects carried the accelerometer for less than 10 h were excluded and the average was calculated on the remaining data, assuming that daily physical activity is an ergodic process. Average counts per day were calculated over the days of measurement. Physical activity level (PAL) was calculated as ratio between TEE and BMR.

### Diurnal activity patterns

Activity counts per minute were aligned according to time of the day. Mean counts over corresponding time intervals of the day for each subject were calculated to create a personalized activity pattern. The diurnal activity pattern was the activity pattern between the average wake-up time and the average go-to-bed time of the subject as derived from the diaries (Wake time). Subsequently, wake time was divided in four quarters of equal time length. This division accounts for potential shifts in the activity pattern, such as the effect of different chronotypes or of work shifts, which are a confounding factor in the study. Activity levels of consecutive quarters were compared to identify diurnal patterns. The first two quarters and the last two quarters were averaged to represent the physical activity of the first and the second half of the day. The difference in physical activity between the first and the second half of the day was a measure of the diurnal drop in physical activity. Sleep time was the time between the individual average go-to-bed time and average wake-up time.

### Aerobic fitness

Aerobic fitness was measured as maximal oxygen uptake during an incremental protocol on a cycle ergometer [29]. After a 5-minutes warming up at a workload of 75W for males and 50W for females, the workload was increased by 50 W every 2.5 min. When the respiratory exchange ratio was higher than one or when heart rate reached a value of 35 bpm below the age-predicted maximal HR (220 bpm—age), the increase in workload was reduced to 25 W until exhaustion. Expired air was collected with a mask covering mouth and nose to be continuously analysed for O2 consumption (Omnical, Maastricht Instruments, Maastricht, The Netherlands). The maximal oxygen uptake was divided by body mass.

### Data analysis

The group was divided in subgroups according to PAL or age. The classification based on PAL defined subgroups of sedentary (PAL<1.75) moderately active (1.75≤PAL<1.90) and active subjects (PAL≥1.90). The classification based on age defined subgroups of adults ≤62 years old, adults 62–≤65 years old, and adults ≥66 years old. Diurnal activity patterns were derived in each subgroup. The significance of the difference between two adjacent quartiles was tested in each subgroup with paired t-test. Comparisons between groups were evaluated with Two-sample t-tests. The Pearson correlation coefficient (r) and the coefficient of determination (r2) were used to describe the association between the diurnal drop in physical activity and aerobic fitness or sleep time. All variables were expressed as mean ± standard deviation (SD). The statistical significance threshold was set at p<0.05.

## Results

Subjects had a PAL between 1.43 and 2.34 (mean±SD, 1.84±0.19) and an aerobic fitness between 18 and 49 ml/kg/min (mean±SD, 29±7 ml/kg/min). Males were fitter than females (33±8 vs. 26±4 ml/kg/min) but with similar PAL, age and wake time. Adults ≤62 years old (N = 10, 5 males, VO2max = 33±6 ml/kg/min) were fitter than adults ≥66 years old (N = 11, 3 males, VO2max = 23±4 ml/kg/min), but with similar PAL and wake time. Sedentary, moderately active and active subjects did not differ in age or wake time but the active group was fitter than the sedentary one (Table 1).

**Table 1:** Subject Characteristics for each Physical Activity Level Group (Mean±SD).

	Sedentary	Moderately Active	Active	Total
Number of subjects	9	11	11	31
Gender (Male/Female)	2/7	7/4	6/5	15/16
Body mass (kg)	73±9	77±11	73±13	74±11
BMI (kg/m^2^)	26.2±1.5	25.9±1.9	25.1±2.7	25.7±2.1
Age (years)	67±8	65±5	61±6	64±6
TEE (MJ/day)	9.27±1.10*	11.26±1.55^¶^	12.22±2.23^¶^	11.02±2.06
BMR (MJ/day)	5.71±0.65	6.19±0.88	6.03±1.12	6.00±0.91s
PAL	1.62±0.09*	1.82±0.04^¶^	2.03±0.14*^¶^	1.84±0.19
Accelerometer assessed activity (kCounts/d)	5.3±1.6	6.0±0.5	7.0±1.6^¶^	6.2±1.4
VO2max (ml/min/kg)	25±5	30±6	32±9^¶^	29±7
Wake time (h/d)	15.5± 1.0	16.0 ±1.0	15.9±0.9	15.9± 1.0

Accelerometer assessed activity, accelerometry output expressed in thousands of counts per day; BMI, body mass index; TEE, total energy expenditure; BMR, basal metabolic rate; PAL, physical activity level calculated as TEE / BMR; VO2max, maximal oxygen uptake per unit of body mass;

*, p<0.05 compared to the moderately active group;

^¶^, p<0.05 compared to the sedentary group.

**Fig 1:**
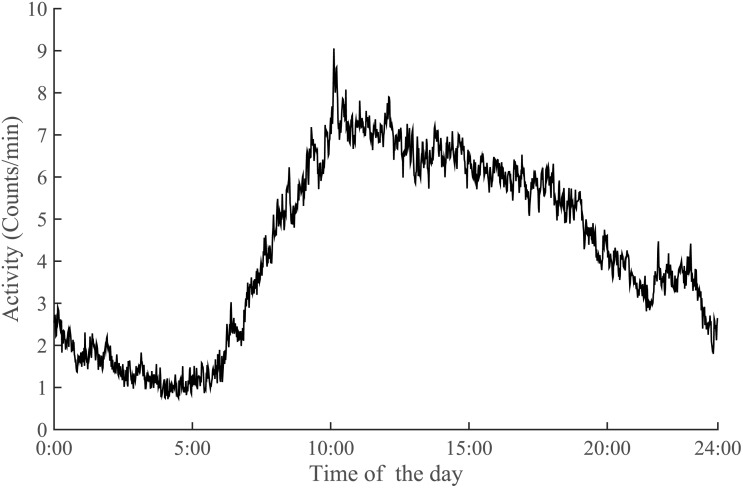
Diurnal physical activity pattern in old subjects. (N = 31).

The 24-hours activity pattern of the group showed a peak of activity in the morning with a subsequent gradual non-linear decrease of about 5% per hour, as represented in Fig 1. All groups reported similar wake and sleep times. Quarters were therefore similar in all groups and went from around 8:00 to around 12:00 (quarter 1, Q1), from around 12:00 to around 16:00 (quarter 2, Q2), from around 16:00 to around 20:00 (quarter 3, Q3), and from around 20:00 to around 00:00 (quarter 4, Q4).

**Fig 2:**
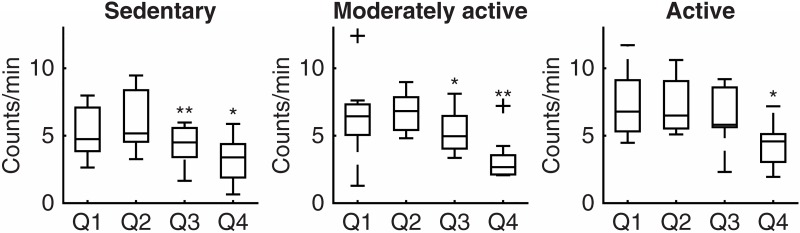
Diurnal activity patterns in sedentary (N = 9, PAL<1.75), moderately active (N = 11, 1.75<PAL<1.90) and active (N = 11, PAL>1.90) older adults. PAL, physical activity level as predicted from daily activity counts; Q1, first quarter of diurnal activity (defined between individuals’ wake-up time and go-to-bed time); Q2, second quarter of diurnal activity (defined between individuals’ wake-up time and go-to-bed time); Q3, third quarter of diurnal activity (defined between individuals’ wake-up time and go-to-bed time); Q4, fourth quarter of diurnal activity (defined between individuals’ wake-up time and go-to-bed time); +, outlier; *, p<0.05 compared to the previous quarter; **, p<0.00505 compared to the previous quarter; ***, p<0.00105 compared to the previous quarter.

**Fig 3:**
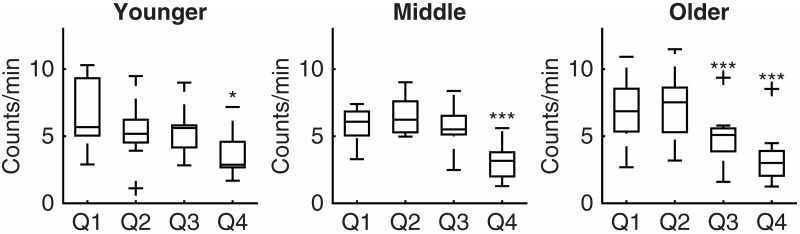
Diurnal activity patterns in adults ≤62 years old (N = 10), adults 62–≤65 years old (N = 10) and adults ≥66 years old (N = 11). Q1, first quarter of diurnal activity defined between individuals’ wake-up time and go-to-bed time); Q2, second quarter of diurnal activity defined between individuals’ wake-up time and go-to-bed time); Q3, third quarter of diurnal activity defined between individuals’ wake-up time and go-to-bed time); Q4, fourth quarter of diurnal activity defined between individuals’ wake-up time and go-to-bed time); +, outlier; *, p<0.05 compared to the previous quarter; **, p<0.00505 compared to the previous quarter; ***, p<0.00105 compared to the previous quarter.

Active subjects reached their peak of activity in Q1, which was maintained until and including Q3 (Fig 2). On average, the activity in this group declined by 11% every quarter. Moderately active and sedentary subjects reached their peak during the Q2 with a significant decrease during Q3 (respectively 29% and 17% drop each quarter). Adults ≤62 years old showed patterns similar to active subjects. Conversely, adults ≥66 years old showed patterns comparable to the sedentary group (Fig 3).

**Fig 4:**
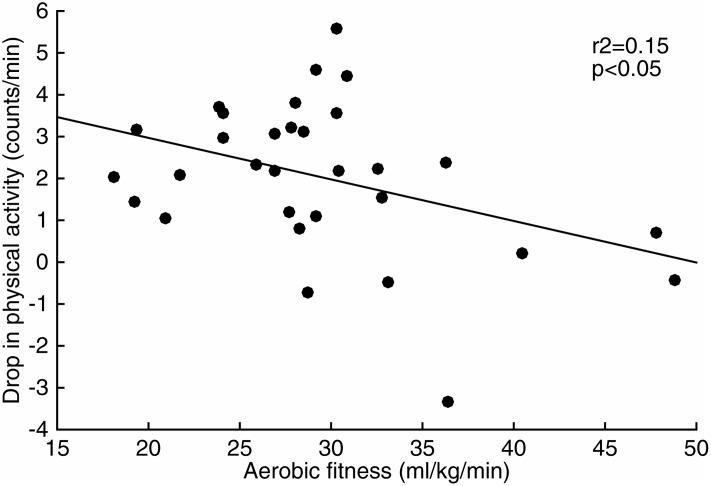
Drop in physical activity between the first and the second half of the day as a function of aerobic fitness in older adults. Aerobic fitness, maximal oxygen intake per kilogram of body mass, r^2^, coefficient of determination.

Fitter subjects showed smaller drops in physical activity between the first and the second half of the day (r = -0.39,p<0.05) (Fig 4). Aerobic fitness, the drop in physical activity, and PAL were not related to wake or sleep time (p>0.5).

## Discussion

This study provides the first evidence that older adults with a lower energy-expenditure derived physical activity level showed a more abrupt drop in physical activity in the afternoon than adults ≤62 years old. Individuals with a more pronounced drop tended to be aerobically less fit.

The PAL range measured in the population of the study was 1.43 to 2.34. Similar PAL ranges are rarely reported in the literature where old subjects rarely show PAL higher than 2.00 [30, 31]. Due to the very active individuals in the group, the population in this study had not only a broad PAL but was also on average very active compared to previous studies [32]. Similarly, the range of aerobic fitness was broader than previous studies [33] and on average subjects were fitter than reported in previous studies, where the average aerobic fitness was around 24ml/kg/min [30]. The broad ranges found in this population allowed to study diurnal activity patterns in active and sedentary as well as in fit and unfit old individuals.

The diurnal activity pattern of the whole group showed a peak around 10AM followed by a progressive decline (Fig 1). This behaviour has been shown before in old individuals, where physical activity declined after a peak between 10:00 and 11:00 to give way to more sedentary behaviours [11, 12]. Comparing the patterns between active and sedentary subjects, active subjects had an earlier peak and sustained their activity level later in the day up to and including the third quarter (16:00–20:00 ca.). These results suggest that old subjects might achieve increased PAL by prolonging active morning bouts of physical activity [11].

One possible explanation for the decrease in physical activity in older adults during wake time is retirement [12]. In the Netherlands, individuals older than 65 y are mostly retired and this might induce a more sedentary lifestyle. However, physical activity declined in all age groups towards the end of wake time (Fig 3). Thus, retirement cannot be the only explanation for the diurnal decline in physical activity. Other explanations include lower aerobic fitness, which is associated with increased fatigue and might affect activity patterns [12, 34]. In the current study, adults ≤62 years old were fitter than adults ≥66 years old and fitter individuals showed a smaller diurnal decline in physical activity (Fig 4). These results support the previously formulated hypothesis that a decrease in physical activity during wake time is mainly determined by aerobic fitness. Further studies might include questions about the occupational status of the subjects to investigate interactions and synergies between aging, physical activity, activity patterns, retirement, and aerobic fitness.

The results of the study are based on the division of the diurnal activity pattern in quarters. A similar method has been proposed before in a study where the physical activity measured during 24 hours was divided in six intervals of four hours starting at fixed times of the day [35]. The method used in that study did not take into account possible shifts in the activity patterns due to individual characteristics such as the chronotype of subjects [36], work shifts, etc. To correct for these individual differences, the current study proposed a division in quarters based on subjects’ specific wake-up and go-to-bed time. This method is applicable only when potential shifts in the activity pattern are a confounding factor rather than an outcome. Wake-up and go-to-bed times were not significantly different between the groups here described, wake and sleep time did not correlate with fitness or the drop in physical activity and the potential shifts in activity patterns were not an outcome of the study. Therefore the division of the diurnal activity pattern in quarters proposed is applicable to this study.

This study shows significant drops in physical activity in the third and fourth quarter of diurnal activity, in spite of the variability of the data. The data presented in Figs 2 and 3 shows different degrees of variability in physical activity between groups and quarters. Future studies including a larger population might provide more detailed results and define whether the variability is a characteristic of the group and quarter or it is due to possible outliers in the population.

Aerobic fitness was shown to be associated with the age-related changes in diurnal activity patterns. However, other physiological factors can be suggested and investigated. In particular altered levels of hormones such as melatonin [37] and cortisol [38] have been reported in individuals with disrupted diurnal patterns, typical of ageing. Both melatonin [37] and cortisol [39] are related to sleep and activity cycles and the disruption of their daily cycle could have a direct effect on diurnal activity patterns. Furthermore, aerobic fitness has been shown to reduce levels of cortisol [40] and increase melatonin in the blood [41]. The study of diurnal activity patterns in subjects with different hormonal profiles could provide further insight into the age-related decline in physical activity and help the development of more effective intervention strategies.

In conclusion, this is the first study to report that active subjects maintain a larger amount of body movement for longer during their wake time. Diurnal physical activity declines more in old adults with lower aerobic fitness. Therefore higher physical activity level might be promoted by prolonging active morning bouts of physical activity and by increasing aerobic fitness.

## Abbreviations

BMI: body mass index
BMR: basal metabolic rate
DLW: doubly labelled water
PAL: physical activity level
Q1: first quarter of diurnal activity
Q2: second quarter of diurnal activity
Q3: third quarter of diurnal activity
Q4: fourth quarter of diurnal activity
TEE: total energy expenditure
VO2max: aerobic fitness measured as maximal oxygen uptake per kilogram of body mass
